# Immune-mediated adverse events in the randomized phase 3 TOPAZ-1 study of durvalumab plus gemcitabine and cisplatin in advanced biliary tract cancer

**DOI:** 10.1093/oncolo/oyaf148

**Published:** 2025-07-07

**Authors:** Lorenzo Antonuzzo, Hidenori Takahashi, Joon Oh Park, Aumkhae Sookprasert, Roopinder Gillmore, Sheng-Shun Yang, Juan Cundom, Mila Petrova, Gina Vaccaro, Marielle Holmblad, Magdalena Żotkiewicz, Julie Wang, Nana Rokutanda, Do-Youn Oh

**Affiliations:** Clinical Oncology Unit, Careggi University Hospital, and Department of Experimental and Clinical Medicine, University of Florence, Largo Brambilla 3, 50134, Florence, Italy; Department of Gastroenterological Surgery, Osaka International Cancer Institute, 3-1-69, Otemae, Chuo-Ku, Osaka, 541-8567, Japan; Division of Hematology-Oncology, Department of Medicine, Samsung Medical Center, Sungkyunkwan University School of Medicine, 81 Irwon-ro, Gangnam-gu, Seoul 06351, South Korea; Medical Oncology Unit, Department of Medicine, Faculty of Medicine, Khon Kaen University, 123 Mittraparp Road, Muang, Khon Kaen 40002, Thailand; Department of Medical Oncology, Royal Free Hospital, Pond Street, London, NW3 2QG, UK; Division of Gastroenterology and Hepatology, Department of Internal Medicine, Taichung Veterans General Hospital, 1650 Taiwan Boulevard, Section 4, Taichung, 40705, Taiwan; Instituto de Investigaciones Metabólicas, Libertad 836, C1012AAR Cdad, Buenos Aires, Argentina; Department of Medical Oncology, MHAT Nadezhda, Blaga Vest Str, 1330 Sofia, Bulgaria; Providence Cancer Institute, 4805 Northeast Glisan Street, Portland, OR 97213, USA; Oncology R&D, Late-Stage Development, AstraZeneca, 1 Medimmune Way, Gaithersburg, MD 20878-2204, USA; Oncology Biometrics, Late Oncology Statistics, AstraZeneca, Postępu 14, 02-676 Warsaw, Poland; Oncology R&D, Late-Stage Development, AstraZeneca, 430 East 29 Street, New York, NY 10016, USA; Oncology R&D, Late-Stage Development, AstraZeneca, 1 Medimmune Way, Gaithersburg, MD 20878-2204, USA; Division of Medical Oncology, Department of Internal Medicine, Seoul National University Hospital, 101 Daehak-ro, Jongno-gu, and Cancer Research Institute, Seoul National University College of Medicine, 103, Daehak-ro, Jongno-gu, Seoul 110-744, South Korea

**Keywords:** Biliary tract neoplasms, gallbladder neoplasms, cholangiocarcinoma, immunotherapy, immune checkpoint inhibitor

## Abstract

**Introduction:**

We assessed immune-mediated adverse events (imAEs) in the TOPAZ-1 (NCT03875235) study of durvalumab plus gemcitabine and cisplatin (GemCis) in advanced biliary tract cancer (aBTC).

**Methods:**

Participants were randomized 1:1 to durvalumab (1500 mg) or placebo, plus GemCis (gemcitabine [1000 mg/m^2^] and cisplatin [25 mg/m^2^]) intravenously, followed by durvalumab (1500 mg) or placebo Q4W. We assessed imAE incidence, time to onset (TTO), and association with overall survival (OS).

**Results:**

In durvalumab (*n* = 338) versus placebo (*n* = 342), imAEs were reported in 13.9% versus 4.7% of participants, with median TTO of 127.0 versus 86.5 days, respectively. OS HR for durvalumab versus placebo in participants with imAEs was 0.59 (95% CI, 0.30-1.23) and was 0.83 (95% CI, 0.70-1.00) in participants without imAEs.

**Conclusions:**

Durvalumab demonstrated an OS benefit versus placebo in aBTC, irrespective of imAEs, which were mostly low grade and manageable. The results in these subgroups were consistent with the overall primary analysis.

**Trial registration:**

ClinicalTrials.gov NCT03875235

## Introduction

Biliary tract cancers (BTCs) account for ~3%–5% of cancers globally and are often only identified when the disease is at an advanced stage.^1^

BTCs may express immune checkpoint proteins, such as programmed cell death ligand-1 (PD-L1),^2^ which has been reported in 59%–68% of people with advanced BTC (aBTC).^3,4^ Immune checkpoint inhibitors (ICIs), including durvalumab, a PD-L1 inhibitor, represent a promising treatment option for aBTC.^3,4^

The randomized, double-blind, global, phase 3 TOPAZ-1 study (NCT03875235) demonstrated significant improvements in overall survival (OS) for durvalumab plus GemCis versus placebo plus GemCis in an interim analysis (stratified OS hazard ratio [HR] from Cox proportional hazards model: 0.80 [95% CI, 0.66-0.97; *P *=.021 (2-sided); significance threshold, 0.030]; data cut-off: August 11, 2021; 59% data maturity) in participants with aBTC, with similar safety between arms.^3^ Subsequently, durvalumab plus GemCis was approved for aBTC.^5^

Although promising, ICIs are associated with immune-mediated adverse events (imAEs): characterized by an excessively activated immune system.^6-8^ Survival benefit with immunotherapy has been observed irrespective of imAE occurrence, with some studies associating imAEs with increased OS.^9^ We assessed imAE incidence and timing, and association between imAE occurrence and OS in TOPAZ-1.

## Methods

### Study design

TOPAZ-1 study details have been described previously.^3^ Briefly, adults with aBTC were randomized 1:1 to durvalumab (1500 mg) or placebo, plus GemCis (gemcitabine [1000 mg/m^2^] and cisplatin [25 mg/m^2^]) intravenously, on a 21-day cycle for up to 8 cycles, followed by durvalumab (1500 mg) or placebo every 4 weeks.

### Assessments

This post-hoc analysis investigated the incidence, timing, and association of imAEs with OS in TOPAZ-1 (data cut-off: February 25, 2022). All imAEs were assessed descriptively and defined as AEs of special or possible interest, associated with drug exposure, consistent with an immune-mediated mechanism of action with no clear alternate etiology. A landmark analysis was conducted to account for immortal time bias. All analyses were exploratory with no control of type I error rate and not powered to determine a statistically significant difference between groups. See Supplementary Material for details.

### Statistical analysis

Safety-related analyses were performed on the safety analysis set (SAS; participants who received ≥1 dose of study treatment). imAEs were analyzed descriptively using summary statistics. Overall survival analyses were performed on the full analysis set (FAS) according to the randomized treatment arm in participants with/without imAEs. Overall survival HR and 95% CI calculations used a Cox proportional hazards model with treatment as the only covariate; medians and their CIs were estimated using Kaplan-Meier.

## Results

The SAS included 680 participants who received ≥1 dose of durvalumab (*n* = 338) or placebo (*n* = 342). The FAS OS analysis for durvalumab and placebo included 341 and 344 participants, respectively.

Overall, 63 participants experienced any-grade imAEs, with proportionally more in participants receiving durvalumab versus placebo (Table 1). The most common imAE in both arms was hypothyroidism; all events were Grade 1/2. Serious imAE incidence was similar between arms. Grade 3 or 4 imAEs were reported in 2.4% versus 1.5% for durvalumab versus placebo, respectively. imAEs leading to discontinuation were comparable between arms. No imAE-related deaths were reported for durvalumab; one death, due to polymyositis, was reported for placebo.

**Table 1.** Summary of imAEs.^a^

	Durvalumab plus GemCis (*n* = 338)	Placebo plus GemCis (*n* = 342)
Participants with any imAE, *n* (%)	47 (13.9)	16 (4.7)
Possibly related to study medication^b^	40 (11.8)	14 (4.1)
Grade 3 or 4	8 (2.4)	5 (1.5)
Serious^c^	6 (1.8)	5 (1.5)
With outcome of death	0	1 (0.3)
Leading to treatment discontinuation^d^	3 (0.9)	4 (1.2)
Median (range) time to onset, days^e^	127.0 (1-511)	86.5 (4-533)
Participants with resolved imAEs, *n* (%)	24 (7.1)	8 (2.3)
Median time to resolution, days (range)^e^	163.0 (1-506^f^)	216.0 (8-415^f^)

^a^Participants may have more than one imAE.

^b^As assessed by the investigator. Missing responses are counted as related.

^c^Seriousness, as assessed by the investigator. An AE with missing seriousness is considered serious.

^d^Includes any AE where the action taken = drug permanently discontinued for at least one treatment.

^e^Includes number of days from first dose to onset of AE.

^f^Censored observation.

Includes AEs with an onset date on or after the date of first dose or pretreatment AEs that increase in severity on or after date of first dose up to and including 90 days following the date of last dose of study medication, or up to and including the date of initiation of the first subsequent therapy (whichever occurs first).

Abbreviations: AE, adverse event; GemCis, gemcitabine and cisplatin; imAE, immune-mediated adverse event; *n*, number of participants.

**Figure 1.**
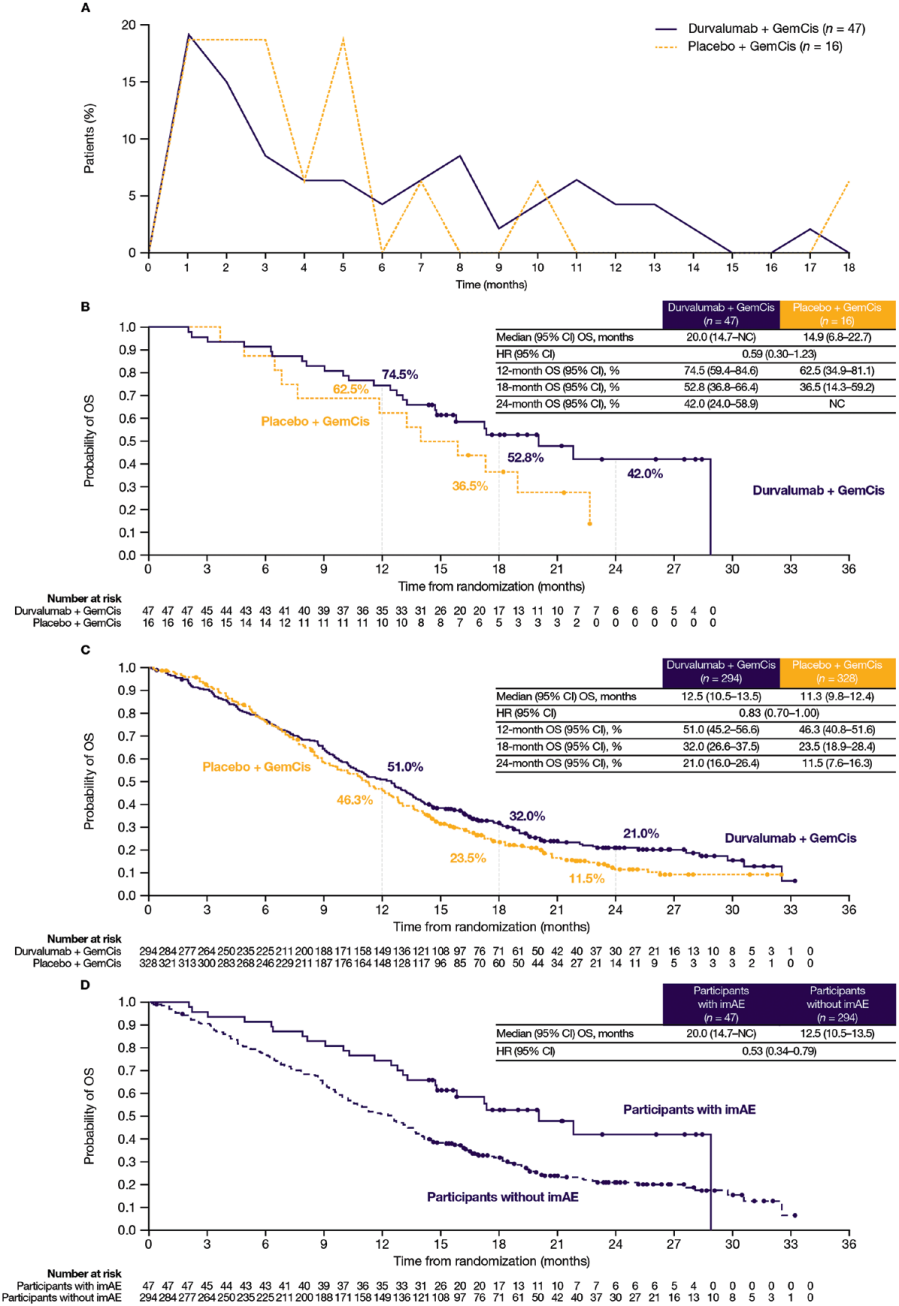
imAE frequency and overall survival in TOPAZ-1 participants. Dots represent censored observations. (A) Overall frequency of imAEs over time for participants treated with durvalumab plus GemCis or placebo plus GemCis. The percentage of participants with an event is the number of participants who experienced ≥1 imAE at each time interval divided by the number of participants who experienced ≥1 imAE at any time; includes first imAE only, regardless of grade. (B) OS in participants with an imAE treated with durvalumab plus GemCis or placebo plus GemCis. (C) OS in participants without imAEs treated with durvalumab plus GemCis or placebo plus GemCis. (D) OS in participants treated with durvalumab plus GemCis by imAE status. Panels B-D: Participants not known to have died at the time of analysis are censored at the last recorded date on which the participant was last known to be alive. Abbreviations: CI, confidence interval; GemCis, gemcitabine and cisplatin; HR, hazard ratio; imAE, immune-mediated adverse event; *n*, number of participants; NC, noncalculable; OS, overall survival. Figure originally presented at the European Society for Medical Oncology Congress 2022 by Lorenzo Antonuzzo et al. Reused with permission.

Time to onset (TTO) of imAEs from first dose ranged from 1 to >500 days, with most occurring within 3 months (Supplementary Table S1, Figure 1A). Steroid and immunosuppressant rescue medications and endocrine therapy were available for participants who experienced imAEs; corticosteroids were the most common treatment in both arms (Supplementary Table S2); >80% of imAEs treated with corticosteroids resolved. imAEs resolved in 7.1% and 2.3% of participants receiving durvalumab and placebo, respectively; most unresolved imAEs for durvalumab were hypothyroidism.

The median (range) duration of follow-up for censored participants with imAEs was 18.3 (14.4-28.5) versus 19.8 (16.4-22.7) months for durvalumab versus placebo, respectively. Overall survival HR for durvalumab versus placebo was 0.59 (95% CI, 0.30-1.23) and 0.83 (95% CI, 0.70-1.00) in participants with and without recorded imAEs, respectively. In participants with and without imAEs, median OS was longer in those treated with durvalumab versus placebo (Figure 1B and C). Overall survival in the durvalumab arm in participants with imAEs versus without is shown in Figure 1D. The landmark analysis demonstrated consistency with these results (Supplementary Figure S1).

## Discussion

In TOPAZ-1, most imAEs in the durvalumab arm were Grade 1/2 and manageable; the most common imAEs were consistent with the known profile of durvalumab and were mainly due to endocrinopathies and skin/subcutaneous tissue events. imAE TTO was consistent with durvalumab in other cancers.^9,10^ In previous ICI reports, gastrointestinal adverse events and skin conditions were among the first imAEs to emerge; endocrinopathies, such as thyroid disorders, generally emerged after 7 weeks.^6^ With durvalumab in TOPAZ-1, maculopapular rash was among the first imAEs to occur, while hypothyroidism generally emerged after 8 weeks, demonstrating TTO consistent with other cancer studies.^6^ The most reported imAE in TOPAZ-1 was hypothyroidism (5.9% with durvalumab), consistent with other ICI studies.^7,10^ As expected in this setting, hypothyroidism in TOPAZ-1 was generally reported as unresolved, though hypothyroidism is typically well-managed with endocrine therapy.^8^

Immunotherapy has demonstrated a positive association between imAEs and improved outcomes.^9^ In TOPAZ-1, OS benefit occurred with durvalumab versus placebo in the FAS^3^; however, participants with imAEs showed numerically greater improvements in OS versus those without imAEs. This effect has been observed across several other cancers.^11^ In this post-hoc analysis, the limited number of participants experiencing imAEs impacted precise estimation of the treatment effect, as demonstrated by wide CIs.

People with aBTC benefit from durvalumab despite the occurrence of imAEs. Therefore, careful monitoring and management of imAEs is important to enable people with aBTC to continue ICI treatment and receive therapeutic benefit. Additional investigations of imAEs could include pathophysiology, management, and diagnostic biomarkers.^7^

This post-hoc analysis demonstrates a consistency of treatment effect with the primary analysis of TOPAZ-1 across participants with or without imAEs,^3^ supporting the use of durvalumab plus GemCis in aBTC and highlighting the importance of imAE management in obtaining maximum treatment benefit.

## Supplementary Material

oyaf148_suppl_Supplementary_Tables_S1-S2_Figures_S1

## Data Availability

Data underlying the findings described in this manuscript may be obtained in accordance with AstraZeneca’s data sharing policy described at https://astrazenecagrouptrials.pharmacm.com/ST/Submission/Disclosure. Data for studies directly listed on Vivli can be requested through Vivli at www.vivli.org. Data for studies not listed on Vivli could be requested through Vivli at https://vivli.org/members/enquiries-about-studies-not-listed-on-the-vivli-platform/. AstraZeneca Vivli member page is also available outlining further details: https://vivli.org/ourmember/astrazeneca/.
