# Supplementary material for: Immune-mediated adverse events in the randomized phase 3 TOPAZ-1 study of durvalumab plus gemcitabine and cisplatin in advanced biliary tract cancer
Source: Oncologist. 2025 Jul 7;30(7):oyaf148. doi: 10.1093/oncolo/oyaf148 (PMC12230790; doi:10.1093/oncolo/oyaf148)
Supplement: oyaf148_suppl_Supplementary_Tables_S1-S2_Figures_S1 [file oyaf148_suppl_supplementary_tables_s1-s2_figures_s1.pdf]

Supplementary material

imAE definition methodology

Immune-mediated adverse events (imAEs) were defined as AEs of special interest or AEs of possible interest, associated with drug exposure, consistent with an immune-mediated mechanism of action with no clear alternate etiology. imAEs were graded by the investigator according to the National Cancer Institute Common Terminology Criteria for Adverse Events v5.0. Reporting included AEs with an onset date on or after the date of first dose or pre-treatment AEs that increased in severity on or after the date of first dose up to and including 90 days following the date of the last dose of study medication, or up to and including the date of initiation of the first subsequent therapy (whichever occurred first). Durvalumab-associated AEs of special interest and AEs of possible interest included Preferred Terms from the Medical Dictionary for Regulatory Activities v24.0 (these Preferred Terms were confirmed prior to the TOPAZ-1 database lock). An automated adjudication process was used to classify imAEs, considering interventions involving systemic steroid therapy, immunosuppressant use, and/or endocrine therapy.

Landmark analysis methodology

A landmark analysis was performed to account for immortal time bias. A time point (landmark) was designated and participants who survived longer than the landmark were included. In this analysis, a 3-month adjusted landmark was applied; all participants censored or with an event within 3 months of randomization were removed.

Figure S1. 3-month landmark analyses of OS in TOPAZ-1 participants. (A) OS in participants with an imAE treated with durvalumab plus GemCis or placebo plus GemCis. (B) OS in participants without imAEs treated with durvalumab plus GemCis or placebo plus GemCis. (C) OS in participants treated with durvalumab plus GemCis by imAE status. All participants censored or with an event within 3 months of randomization were removed. Participants not known to have died at the time of analysis were censored at the last recorded date on which the participant was last known to be alive. Dots represent censored observations. Abbreviations: CI, confidence interval; GemCis, gemcitabine and cisplatin; HR, hazard ratio; imAE, immune-mediated adverse event; *n*, number of participants; NC, non-calculable; OS, overall survival.

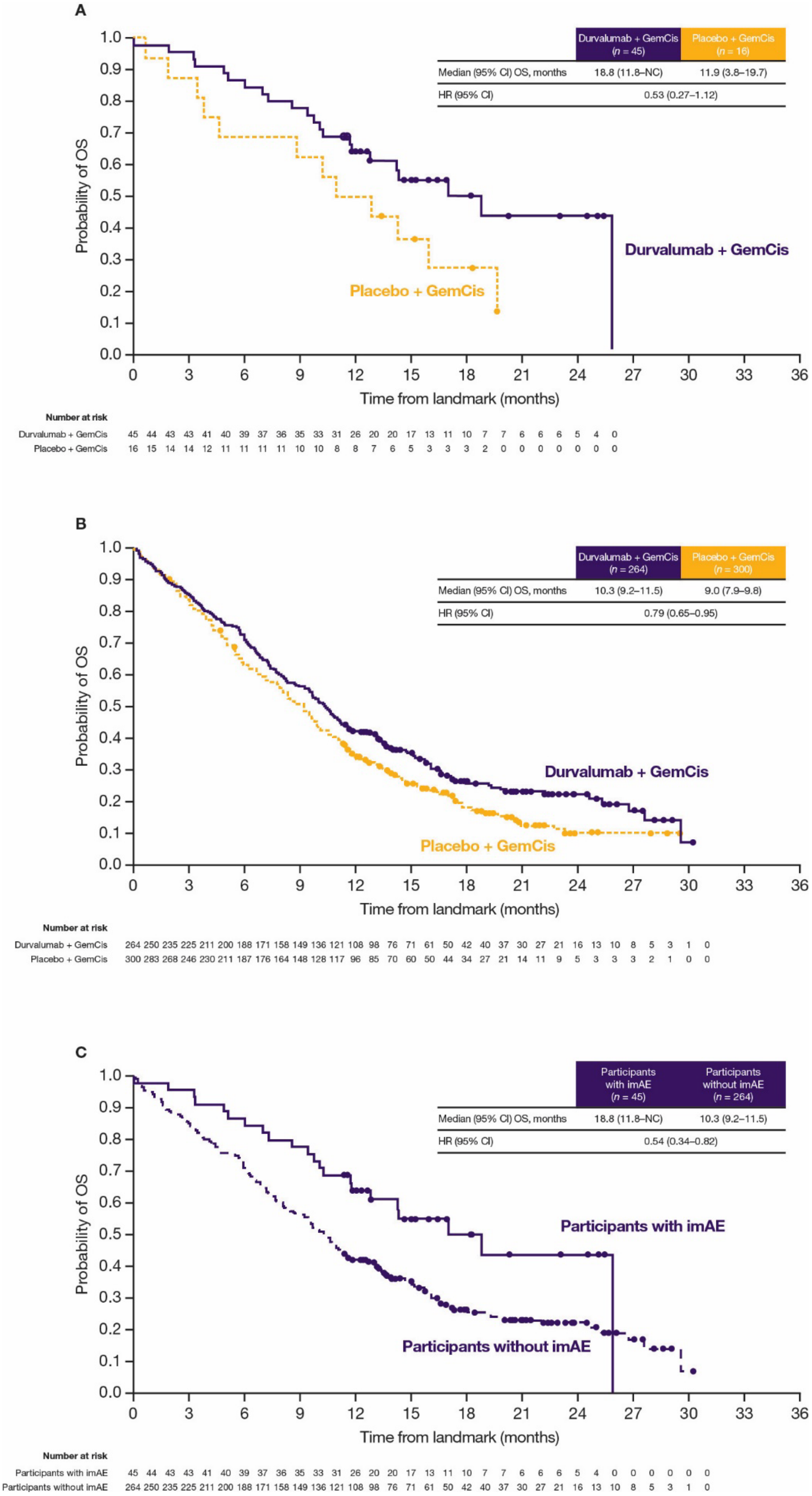

A
Durvalumab + GemCis (n = 45)
Placebo + GemCis (n = 16)
Median (95% CI) OS, months 18.8 (11.8–NC) 11.9 (3.8–19.7)
HR (95% CI) 0.53 (0.27–1.12)
Probability of OS
Durvalumab + GemCis
Placebo + GemCis
Time from landmark (months)
Number at risk
Durvalumab + GemCis 45 44 43 43 41 40 39 37 36 35 33 31 26 20 20 17 13 11 10 7 7 6 6 6 5 4 0
Placebo + GemCis 16 15 14 14 12 11 11 11 11 10 10 8 8 7 6 5 3 3 3 2 0 0 0 0 0 0 0
B
Durvalumab + GemCis (n = 264)
Placebo + GemCis (n = 300)
Median (95% CI) OS, months 10.3 (9.2–11.5) 9.0 (7.9–9.8)
HR (95% CI) 0.79 (0.65–0.95)
Probability of OS
Durvalumab + GemCis
Placebo + GemCis
Time from landmark (months)
Number at risk
Durvalumab + GemCis 264 250 235 225 211 200 188 171 158 149 136 121 108 98 76 71 61 50 42 40 37 30 27 21 16 13 10 8 5 3 1 0
Placebo + GemCis 300 283 268 246 230 211 187 176 164 148 128 117 96 85 70 60 50 44 34 27 21 14 11 9 5 3 3 3 2 1 0 0
C
Participants with imAE (n = 45)
Participants without imAE (n = 264)
Median (95% CI) OS, months 18.8 (11.8–NC) 10.3 (9.2–11.5)
HR (95% CI) 0.54 (0.34–0.82)
Probability of OS
Participants with imAE
Participants without imAE
Time from landmark (months)
Number at risk
Participants with imAE 45 44 43 43 41 40 39 37 36 35 33 31 26 20 20 17 13 11 10 7 7 6 6 6 5 4 0 0 0 0 0 0
Participants without imAE 264 250 235 225 211 200 188 171 158 149 136 121 108 98 76 71 61 50 42 40 37 30 27 21 16 13 10 8 5 3 1 0

Supplementary Table S1. Incidence of imAEs and median time to onset of imAEs with incidence > 0.5% in either treatment arm

	**Durvalumab plus GemCis (*n* = 338)**		**Placebo plus GemCis (*n* = 342)**	
**imAEs and mTTO (days)**	**Any grade**	**Grade 3 or 4**	**Any grade**	**Grade 3 or 4**
Hypothyroidism, *n* (%)	20 (5.9)	0	5 (1.5)	0
mTTO,[a] days (Q1–Q3)	132.5 (61.0–267.0)	–	98.0 (45.0–202.0)	–
Adrenal insufficiency, *n* (%)	4 (1.2)	0	1 (0.3)	0
mTTO,[a] days (Q1–Q3)	206.0 (96.0–408.5)	–	143.0 (143.0–143.0)	–
Hyperthyroidism, *n* (%)	2 (0.6)	0	0	0
mTTO,[a] days (Q1–Q3)	46.0 (42.0–50.0)	–	–	–
Maculopapular rash, *n* (%)	6 (1.8)	3 (0.9)	0	0
mTTO,[a] days (Q1–Q3)	16.5 (4.0–37.0)	–	–	–
Rash, *n* (%)	4 (1.2)	0	1 (0.3)	0
mTTO,[a] days (Q1–Q3)	203.0 (148.0–351.5)	–	134.0 (134.0–134.0)	–
Pruritus, *n* (%)	2 (0.6)	0	0	0
mTTO,[a] days (Q1–Q3)	240.5 (137.0–344.0)	–	–	–
Immune-mediated hepatitis, *n* (%)	2 (0.6)	1 (0.3)	0	0

mTTO,[a] days (Q1–Q3)	28.0 (7.0–49.0)	–	–	–
Pneumonitis, *n* (%)	2 (0.6)	1 (0.3)	2 (0.6)	1 (0.3)
mTTO,[a] days (Q1–Q3)	270.0 (11.0–529.0)	–	47.0 (15.0–79.0)	–
Diarrhea, *n* (%)	3 (0.9)	1 (0.3)	1 (0.3)	1 (0.3)
mTTO,[a] days (Q1–Q3)	134.0 (80.0–342.0)	–	8.0 (8.0–8.0)	–

[a]Includes number of days from first dose to onset of AE.

Includes AEs with an onset date on or after the date of first dose or pre-treatment AEs that increase in severity on or after date of first dose up to and including 90 days following the date of last dose of study medication, or up to and including the date of initiation of the first subsequent therapy (whichever occurs first). mTTO was not calculated for Grade 3 or 4 imAEs.

Abbreviations: AE, adverse event; GemCis, gemcitabine and cisplatin; imAE, immune-mediated adverse event; mTTO, median time to onset; *n*, number of participants; Q, quartile.

Supplementary Table S2. Treatment of imAEs.

	**Durvalumab plus GemCis (*n* = 338)**	**Placebo plus GemCis (*n* = 342)**
Participants with any imAE, *n* (%)	47 (13.9)	16 (4.7)
Received systemic corticosteroids	29 (8.6)	12 (3.5)
Received high-dose steroids	14 (4.1)	10 (2.9)
Received endocrine therapy	24 (7.1)	5 (1.5)
Received other immunosuppressants	1 (0.3)	1 (0.3)

Abbreviations: GemCis, gemcitabine and cisplatin; imAE, immune-mediated adverse event; *n*, number of participants.
